# Partnership for European Research in Occupational Safety and Health (PEROSH) – Celebrating 20 years of collaboration

**DOI:** 10.5271/sjweh.4110

**Published:** 2023-09-01

**Authors:** Steffen Bohni Nielsen, Margrethe Schøning, Louis Laurent, Jan Michiel Meeuwsen

**Affiliations:** NFA, Denmark.; STAMI, Norway; INRS, France; PEROSH, The Netherlands.

In September 2023, researchers and research directors from the Partnership for European Research in Occupational Safety and Health (PEROSH) convene at its conference in Stockholm. The conference marks the 20^th^ anniversary of European collaboration. PEROSH is comprised of 14 research institutes from 13 European countries: the Austrian Social Insurance for Occupational Risks (AUVA), the (German) Federal Institute for Occupational Safety and Health (BAuA), the (Polish) Central Institute for Labour Protection - National Research Institute (CIOP-PIB), the Finnish Institute of Occupational Health (FIOH), the (UK) Health and Safety Executive (HSE), the Italian Workers Compensation Authority (INAIL), the (German) Institute for Occupational Safety and Health (IFA), the (French) National Institute of Occupational Research and Risk Prevention (INRS), the (Spanish) National Institute of Safety and Health at Work (INSST), the (Danish) National Research Centre for the Working Environment (NFA), the Swedish Agency for Work Environment Expertise (SAWEE), the (Norwegian) National Institute of Occupational Health (STAMI), the (Dutch) Organisation for Applied Scientific Research (TNO), and the (Swiss) Center for Primary Care and Public Health (Unisanté). Together, PEROSH's member institutes comprise more than 2000 occupational safety and health (OSH) experts and provide a significant research contribution to OSH research in Europe and globally. In a study of six OSH networks from different parts of the world, the International Labor Organization (ILO) identified that PEROSH had “strong national and regional policy impact” and profiled the partnership in detail (1, 2).

## The history of PEROSH

The establishment of PEROSH was inspired by the continued relevance of the international “Sheffield Group”, an informal network of directors general of national OSH research institutes across the globe. The network first met in 1989 at the laboratory of the HSE (at that time based in Sheffield). Its members span four continents: North America (IRSST – Canada and NIOSH – USA); Oceania (NIOH –New Zealand), Asia (IIOSH – Israel, JNIOSH – Japan, KOSHA – South Korea and WSHI – Singapore), and Europe. The institutes host annual meetings on a rotating basis. After the turn of the millennium, European members realized the need to address OSH transnational issues at the European level. National institutes for OSH research in Europe play a distinctive role in identifying salient or emerging OSH issues, producing, and synthesizing evidence to inform decisions for a plethora of system actors such as national policymakers, regulators, health and insurance systems, and industry and trade unions. In an increasingly globalized world, OSH challenges transcend national borders and the European Union continues to play an important role. European members recognized the opportunity to exploit role synergies and leverage national funding through collaboration. This opportunity initiated the establishment of PEROSH.

## Structure and organization

The partnership operates through a formal agreement renewed in 2018 and is formally governed by a Steering Committee (SC), consisting of the directors general or research directors of each member institute. The SC is responsible for the strategy and management of PEROSH, including financial decisions and admitting new members to its ranks. Further, the Executive Committee supports the SC in managing operations. The Scientific Steering Group, which comprises the Research Director or Head of Science for each institute, sets priorities, and manages all joint research activities and events.

Each member institute pays an annual membership fee. The fees fund support activities, including the Manager of International Affairs (MIA), the Secretariat, digital resource management and dissemination.

## Mission and vision

The mission of PEROSH is to create and exchange new research-based OSH knowledge and practice and make it *accessible* to policymakers, organizations, employees, and employers in the worklife and other stakeholders in Europe for better decision-making in policy and practice (3). PEROSH’s vision is that policymakers, organisations, employees and employers in the worklife and other stakeholders across Europe are informed by and use PEROSH-generated knowledge in their efforts to develop innovative and sustainable, healthy and safe workplaces.

The partnership seeks impact and adds value through the exchange of knowledge and good practices across Europe to the benefit of all its members and stakeholders. The PEROSH strategy outlines four areas of activity to attain its mission: (i) pooling resources and create new OSH evidence by joint activities; (ii) disseminating and exchanging knowledge on OSH issues between members and to relevant stakeholders nationally and European institutions; (iii) providing evidence that informs decisions by national and European policymakers on healthy and safe working lives; and (iv) identifying emerging research challenges in the OSH field.

## Research priorities

The Joint Research Programme was introduced in 2009 and aimed at realizing synergies in distinctive research needs that transcend national boundaries across the European institutes. Today, PEROSH’s Joint Research Programme consists of a set of collaborative activities (research projects, exploratory workshops, scientific conferences etc.) between member institutes driven by member institutes’ research priorities. Around 6–10 activities are ongoing every year. Normally, about half of the 14 partner institutes are involved in each one. Some projects also include research institutes outside PEROSH.

Many projects address the need to both facilitate and undertake the synthesis of existing research evidence. Across the PEROSH collaboration projects, cross-cutting aims include:

Generating or synthesizing evidence that can be used at national level to underpin and inform decision-making at policy, intermediary, and workplace levels;
Collective development of practical tools and guidelines for use by OSH professionals and companies to support occupational health and safety;
Development of data review methodologies, taxonomies and databases to enable national-level research and survey data to be captured and used systematically for transnational evidence synthesis; and
Comparisons of national-level health and safety practices and guidelines to identify new avenues for exploration by the wider research community to deliver common needs for Europe.

A few years ago, an interdisciplinary group of researchers from PEROSH member institutes conducted a review of European research priorities. The research team applied a modified Delphi study, which was a first attempt to provide the European OSH research community perspectives on priorities for further OSH research. They noted differences across European geographical areas. Overall the most important research topics identified were: older workers; nanomaterials; emerging technological devices; chemical agents; and working conditions, working organisation and job content. Gauging across research priorities variations, priority was also given to emergent issues such as OSH management in new forms of employment (ie. precarious work); impacts of innovation and new ways of working (ie, telework, boundary less work); health and safety in human–computer interaction (ie, artificial intelligence); and the introduction of unsafe and unhealthy work equipment.

Further, two transversal priorities were identified: knowledge translation and exchange of research results into practical and effective tools; and supporting implementation of OSH research findings in micro, small and medium enterprises (4). Some of these priorities are reflected in current joint research projects in PEROSH.

Over the years, the collaboration in PEROSH has resulted in more than 40 joint research publications, several researcher exchange programs, development of methodology guidelines, establishment of databases, position papers, and advocacy towards the European Commission and Parliament on research priorities in OSH policy development. For PEROSH and its member institutes, it remains a continuous priority to identify and utilize pathways to influence OSH policy decisions at European level and at national level.

## Knowledge dissemination

Outcomes of PEROSH’s joined activities are disseminated through its website, social media (Twitter, LinkedIn), newsletters and webinars. Special PEROSH sessions were organized at the last World Congress for Safety and Health and ICOH Congress. PEROSH has a publicly accessible repository with all its report, articles, and presentations. This repository can be disclosed using any search term one prefers. Policy-makers at the European level are reached through personal contacts with the European Commission and its agencies and through the distribution of fact sheets. A close link is established with the Commission's Directorate General for Employment on guidance issues and the EU Agency for Occupational Safety and Health (EU-OSHA) on research priorities. Policy-makers on the national level are reached through the individual members. Prolonging Working Life, research into how prolonged working life is tackled in Europe, was initiated on a request from the Danish Government and NFA, the Danish PEROSH member, who sought to use the PEROSH network to see other countries deal with this issue.

## Conclusion

Despite policy and practical efforts throughout Europe, occupational exposures resulting in health and safety challenges remains a considerable burden for the individual, society and the economy (5). If the challenges are to be met, it is essential to leverage scarce research funds, exchange knowledge and improve the evidence base for political priorities. Over the past 20 years, PEROSH has become an important platform for leveraging discussions and providing input to policymakers at the European level.
